# Food Proteins as Functional Ingredients in the Management of Chronic Diseases: A Concise Review

**DOI:** 10.3390/nu16142323

**Published:** 2024-07-19

**Authors:** Thaniyath Shahnaz, Abosede O. Fawole, Adeyemi A. Adeyanju, John O. Onuh

**Affiliations:** 1 Department of Food and Nutritional Sciences, Tuskegee University, 1200 W. Montgomery Rd, Tuskegee, AL 36088, USA; tshahnaz1460@tuskegee.edu; 2 Biology Department, The Polytechnic, Ibadan 200132, Nigeria; fawole.abosede@polyibadan.edu.ng; 3 Department of Food Science and Microbiology, Landmark University, PMB 1001, Omu-Aran 251103, Nigeria; adeyanju.adeyemi@lmu.edu.ng

**Keywords:** dietary, proteins, bioactive peptides, cardiovascular diseases, chronic diseases, nutritional

## Abstract

Chronic diseases have emerged as a formidable global health concern, with their prevalence steadily rising over the years. Several approaches to addressing these concerns include the use of medications, which are often expensive, contain synthetic chemical substances, and have reported adverse effects. The use of foods, especially proteins, as an alternative approach to addressing chronic health concerns by treating and managing chronic diseases is increasing. This review evaluates the intriguing role of food proteins in mitigating chronic diseases and improving our understanding of the therapeutic potential of different protein types, including those derived from legumes, nuts, and seeds, dairy, fish, and numerous other sources. They have been reported to offer promising avenues for managing chronic diseases, including cardiovascular diseases, diabetes, chronic inflammation, weight management, bone health, glycemic control, muscle preservation, and many other health benefits. Although the exact mechanisms for these actions are still not properly elucidated, it is, however, understood that food proteins exert these health-beneficial effects by their unique nutritional and bioactive profiles, especially their bioactive peptides and amino acids. Practical applications are also discussed, including dietary interventions that are tailored towards incorporating protein-rich foods and the development of functional foods for disease prevention and management. Food proteins are a promising approach to combating chronic diseases that can turn around public health practices.

## 1. Introduction

Chronic diseases remain a significant global health concern, with their prevalence now extending to regions where they were previously uncommon. These diseases, which include heart disease, diabetes, obesity, cancer, neurodegenerative disorders, metabolic disorders, and a host of other diseases, pose a great health challenge [1]. The importance of these chronic diseases is emphasized by their prevalence among the vulnerable population, which accounts for about 71% of all deaths, according to the World Health Organization [2]. Several approaches to addressing these concerns include the use of medications, which are often expensive and contain synthetic chemical substances that have been reported to have some adverse effects. One aspect that is often overlooked is the link between these chronic diseases and dietary patterns, including protein intake, considering that nutrient intake is reported to be a modifying factor in communities with a significant prevalence of these disease outcomes [3]. The trend of employing dietary elements, particularly food proteins, as an alternative strategy for tackling chronic health issues and managing chronic diseases is rising [4].

The kind and amount of protein consumed have a tremendous impact on the development, prevention, management, and control of diseases. For instance, consuming proteins, especially from red and processed meats, has been associated with a higher chance of colorectal cancer and cardiovascular diseases (CVD) [5]. These associations highlight how our dietary choices influence our health outcomes [6]. It is also worth noting that the source of proteins matters a lot, as plant-based proteins from legumes, nuts, and seeds have been linked to reduced risk factors for chronic diseases [7]. On the other hand, insufficient consumption of proteins among those who are more susceptible can lead to malnutrition and a weakened immune system, thereby worsening the impact of long-term illnesses [8]. Moreover, consuming sufficient proteins becomes more crucial as people age, which is vital for maintaining muscle strength and overall well-being [9].

Hence, in the wider conversation about worldwide well-being, acknowledging the complex relationship between protein intake and long-term illnesses is crucial for developing successful prevention and management tactics [1]. Tackling eating habits, advocating for diverse protein sources, and customizing protein guidelines based on different groups of people are vital elements of a holistic method to reduce the global impact of chronic diseases [10]. Modern tools, including nutrigenomics, nutrigenetics, metabolomics, and t other omics, can be of great benefit as discussions shift more towards personalized nutrition and health. 

**Figure 1:**
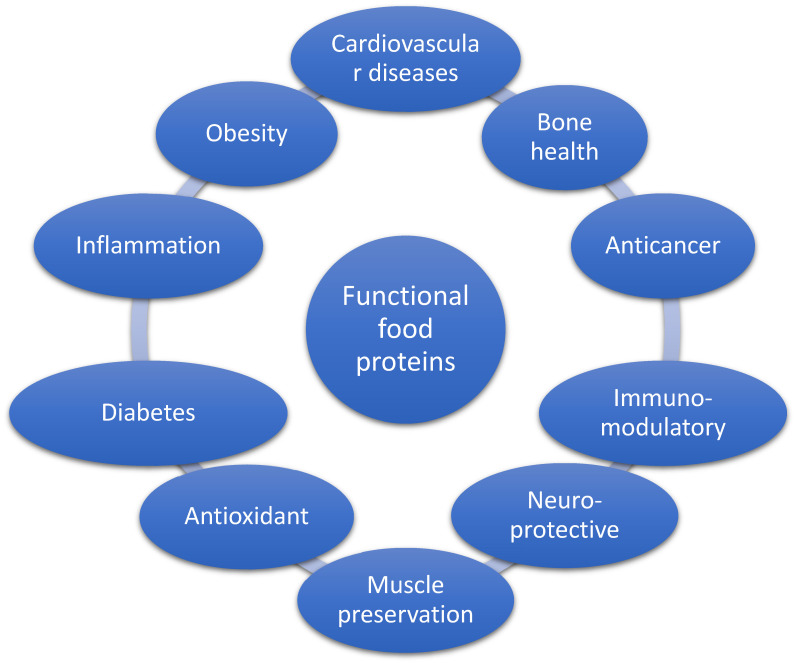
Functional food proteins and chronic disease management.

Although they have been reported to offer promising avenues for managing chronic diseases, including CVD, diabetes, chronic inflammation, weight management, bone health, glycemic control, muscle preservation, and so many other health benefits (Figure 1), the exact mechanisms for their actions, however, are still not properly elucidated. Food proteins are believed to exert their health benefits by virtue of their unique nutritional and bioactive profiles, especially their bioactive peptides and amino acids. Therefore, this review aims to evaluate the intriguing role of food proteins in mitigating chronic diseases and improving our understanding of the therapeutic potentials of different protein types, including those derived from legumes, nuts, and seeds, dairy, fish, and numerous other sources.

## 2. Chronic Diseases and Dietary Influence

Chronic diseases, or non-communicable diseases, are long-lasting conditions that typically progress slowly over time and can persist for years or a lifetime. There are several common chronic diseases that have a significant impact on an individual’s health and well-being. CVD, such as hypertension and coronary artery disease, are leading cause of morbidity and mortality worldwide [9]. Respiratory conditions like chronic obstructive pulmonary disease and asthma can severely affect lung function [10]. Diabetes, both type 1 and type 2, leads to high blood sugar levels and can result in various complications [11]. Additionally, chronic diseases include cancer, a diverse group of conditions characterized by the uncontrolled growth of abnormal cells [12]. Other prevalent chronic diseases encompass neurological disorders like Alzheimer’s disease and Parkinson’s disease, as well as autoimmune diseases such as rheumatoid arthritis and multiple sclerosis [13]. Managing chronic diseases often involves long-term medical care, lifestyle modifications, and regular monitoring to mitigate their impact on an individual’s quality of life.

Chronic disease management is a critical aspect of modern healthcare, as chronic conditions are responsible for a substantial portion of the global disease burden. Effective management of chronic diseases is essential not only for improving the quality of life of individuals living with these conditions but also for reducing healthcare costs and preventing complications that can lead to disability and premature mortality [11,14]. Diet plays a pivotal role in managing chronic diseases, with a particular emphasis on protein intake. Protein is an essential nutrient that serves numerous bodily functions, and its role in chronic disease management is multifaceted. Whether it is managing conditions like diabetes, cardiovascular diseases, or kidney disease, the right protein diet can significantly impact patient outcomes [15].

For individuals with diabetes, managing blood sugar levels is a primary concern. Protein can play a crucial role in this by helping to stabilize blood sugar. Unlike carbohydrates, which can cause rapid spikes in blood sugar levels, protein has a slower and more sustained effect [16]. Incorporating lean protein sources, such as poultry, fish, beans, and tofu, into one’s diet can help prevent sharp increases in blood sugar after meals [17]. This is especially important for individuals with diabetes to maintain glycemic control [18]. In the context of cardiovascular diseases, managing risk factors like high blood pressure and cholesterol levels is paramount. Lean and plant-based protein sources are typically lower in saturated fats and cholesterol, which helps manage risk factors associated with CVD, including high blood pressure and elevated cholesterol levels. Maintaining a balanced diet that includes an appropriate amount of protein alongside other essential nutrients is crucial, emphasizing whole foods and minimizing processed or red meats [19]. 

For individuals with kidney disease, protein intake is often a point of concern [8]. The Kidney plays a vital role in filtering waste products from protein metabolism. In cases of impaired kidney function, there may be a need to limit protein intake to reduce the kidney’s workload [17]. A diet with high-quality protein sources, such as eggs and poultry, and controlled quantities of protein can be recommended to manage kidney disease and prevent further damage [7]. Furthermore, protein is essential for muscle maintenance and repair, which is vital for overall health, particularly as people age [20]. Many chronic diseases can lead to muscle wasting or weakness, so ensuring an adequate protein intake can help maintain muscle mass and strength. It is important to note that the type of protein matters as well. Lean and plant-based protein sources are generally considered healthier choices. Reducing the consumption of processed and red meats, which are associated with an increased risk of chronic diseases, is often advised [5,21]. The overall balance of macronutrients in the diet, including carbohydrates and fats, should also be considered when developing a dietary plan for chronic disease management.

## 3. Recent Developments on the Potential Impact of Dietary Proteins in the Management of Chronic Diseases 

The importance of food proteins in the management of chronic diseases has gained increasing attention within the global health and nutrition sciences field in recent years. Chronic diseases are highly prevalent and require comprehensive approaches for their prevention, management, and treatment. Recent studies indicate that food proteins, which possess various properties and bioactive components, could significantly impact in this regard [22,23,24]. For example, specific proteins derived from dairy products have been associated with regulating blood pressure and may contribute to controlling hypertension or high blood pressure, a major risk factor for CVD [25]. Moreover, plant-based proteins found in legumes and soybeans have shown promise in enhancing glycemic control, thereby offering valuable support in managing diabetes and its associated health complications and comorbidities [26].

In addition, protein consumption plays a crucial role that goes beyond its nutritional benefits. Certain proteins found in foods possess bioactive peptides that possess antioxidant, antihypertensive, anti-diabetic, anti-obesity, anti-inflammatory, immune-modulatory, and several other bioactive properties [27]. These properties can greatly contribute to improving the outcomes of chronic diseases and the quality of health and lives among vulnerable populations. Moreover, the extent to which protein sources are absorbed and digested affects nutrient absorption rates significantly [28]. This is particularly important for older adults who may face challenges related to protein malnutrition and for infants suffering from protein-energy malnutrition.

Exploring the intricate connection between proteins found in foods and long-term illnesses presents exciting possibilities for customized dietary interventions. This places significant emphasis on personalized dietary strategies that consider various factors like genetic inclination, cultural eating habits, and overall nutritional well-being [29,30,31,32]. The possibility for food proteins to play a role in managing chronic diseases necessitates additional research efforts to unravel other opportunities that may not previously have been known. When applied together with broader approaches that promote health and lifestyle modifications, this opportunity creates the potential for advancing global health outcomes towards better improvements and transforming lives [33].

In addition to their direct impact on chronic diseases, dietary proteins can also be used to address the complex link between nutrition and health. The food is consumed primarily for nutrition, but the association between these nutrients and health outcomes is blurry in some cases, with their mechanisms still unexplained [34]. For individuals with health conditions such as celiac disease or gluten sensitivity, finding alternative sources of protein is paramount to preventing unwanted health outcomes [35]. Plant-based proteins, like pea proteins and quinoa proteins, make it easier for people who need to follow a gluten-free diet to find what they need. This shows that edible proteins can be changed to fit the needs of people who have digestive problems or allergic reactions to these types of proteins [36]. Also, emerging research suggests that protein supplementation throughout the day, known as protein pacing, may play a role in regulating muscle protein synthesis, thereby reducing muscle mass in older adults and associated weight loss, all of which are closely associated with chronic disease outcomes [23]. These findings highlight the importance of quantity, timing, quality, and source of dietary proteins in diseases without emphasizing their position [37]. However, it is important to recognize that the potential benefits of dietary protein depend on a holistic approach to nutrition, especially personalized nutrition. Nutrient interactions, including those between proteins and other metabolites found in whole foods, and our genetic makeup must be considered [38]. In addition, promoting a balanced, sustainable, and culturally sensitive diet is important for successfully integrating dietary proteins into chronic disease management strategies [39].

Dietary proteins offer multifaceted tools for managing chronic diseases, especially CVD, which is a leading risk factor for death in the US and many Western societies [40]. Their ability to meet specific healthcare needs, support vascular health, and enhance overall nutrition holds promise for individuals and populations [41]. However, as with any nutritional intervention, an individualized approach and evidence-based research are very important in order to realize the full potential of dietary proteins for the general population, especially populations that are critically at risk of these chronic diseases.

## 4. Mechanisms of Action of Food Proteins in the Management of Chronic Diseases 

Chronic diseases are complex conditions with multifaceted mechanisms of action that contribute to their development and progression [42]. The role of a protein-rich diet in the modulation of chronic diseases is crucial and can be understood through various mechanisms and pathways. Protein intake can play a significant role in chronic disease modulation, particularly in relation to diabetes, CVD, cancer, neurodegenerative diseases, inflammation, kidney diseases and several other conditions [17]. In the case of diabetes, a protein-rich diet can help regulate blood sugar levels, modulate oxidative stress by quenching generated free radicals in adipose tissues, regulate satiety by creating a sense of fill as well as encourage sugar uptake by other tissues [43]. Proteins have a slow and sustained impact on blood sugar, preventing rapid spikes and facilitating better glycemic control. By incorporating lean protein sources like poultry, fish, beans, and tofu into the diet, individuals with diabetes can better manage their condition [21].

Protein can also be instrumental in managing risk factors for CVD, especially by the action of hydrolyzed peptides which play a major role in regulating the renin angiotensin aldosterone systems (RAAS) and also control oxidative stress [44]. Certain protein sources, such as fatty fish like salmon and trout, are rich in omega-3 fatty acids. Omega-3 fatty acids have been linked to reduced heart disease risk by decreasing inflammation and improving heart health. Furthermore, a diet that prioritizes lean protein over red meat and processed meats can help reduce saturated fat intake associated with heart disease [16]. This has the effect of improving overall heart health and preventing incidences of CVD and CV mortality [14,16,44].

In cases of kidney disease, protein intake is a key consideration, especially, the quality and quantity of proteins consumed [5]. The kidneys are responsible for filtering waste products from protein metabolism. If kidney function is compromised, it may be necessary to limit protein intake to reduce the strain on the kidneys [19,29]. In this situation, a diet that focuses on high-quality protein sources, such as eggs, poultry, and controlled protein quantities, may be recommended to effectively manage kidney disease and prevent further damage [45]. One of the mechanisms by which the food proteins modulate kidney health is through regulating oxidative stress by acting as an antioxidant and quenching generated free radicals [14].

Protein is also essential for muscle maintenance and repair, which is vital for overall health, particularly as individuals age [46]. Chronic diseases can often lead to muscle wasting or weakness, otherwise referred to as muscle atrophy. Maintaining adequate protein intake can help preserve muscle mass and strength, which is crucial for mobility and overall health [5,47]. The quantity and quality of proteins, especially in terms of the quantities of essential amino acids, is very crucial for muscle strength and health. This also promotes increased body protein turnover with resultant benefits on muscular tissues. It is important to highlight the types of protein sources in the diet. Emphasizing lean and plant-based protein sources over red meat and processed meat is generally considered healthier. Reducing the consumption of processed and red meats can help lower the intake of saturated fats and potentially reduce the risk of certain types of cancer [28]. This leads to reduced oxidative stress and inflammation with improved overall health.

Bioactive peptides, derived from various sources, including dietary proteins, have gained significant attention for their potential anti-inflammatory effect in addition to their known antihypertensive, antioxidative, antidiabetic, anticholesterolemic, immunomodulatory, anticarcinogenic and other beneficial health effects [48]. These anti-inflammatory peptides can modulate cytokine production, which is a critical component of the body’s immune response and inflammatory processes [49]. Bioactive peptides exert their anti-inflammatory influence through several mechanisms. They may inhibit the activation of pro-inflammatory transcription factors, such as nuclear factor-kappa B (NF-κB). This inhibition prevents the expression of pro-inflammatory genes and the subsequent production of cytokines like tumor necrosis factor-alpha (TNF-α) and interleukin-6 (IL-6). Additionally, bioactive peptides can directly bind to specific receptors on immune cells, thus interfering with the signaling pathways that lead to cytokine release [50]. Furthermore, some bioactive peptides possess antioxidant properties. By reducing oxidative stress and scavenging free radicals, they mitigate the activation of inflammatory pathways, thereby decreasing cytokine production. These peptides can also stimulate the production of anti-inflammatory cytokines, such as interleukin-10 (IL-10), which counterbalance the pro-inflammatory response [51].

## 5. Diverse Functions of Proteins 

Proteins comprised of amino acids and play a pivotal role in the human body’s intricate web of biological processes. Serving as catalysts, enzymes like catalase facilitate chemical reactions, safeguarding cells from oxidative stress. Structural proteins such as collagen contribute to the integrity of connective tissues, providing strength to vital structures like skin and tendons. Hemoglobin, a transport protein, ferries oxygen through the bloodstream, ensuring the energy needs of tissues and organs are met. Antibodies, like immunoglobulin G (IgG), function as defenders, recognizing and neutralizing pathogens. Beyond these examples, the diverse functions of proteins span cellular signaling, immune response, and metabolic regulation, emphasizing their indispensable nature in maintaining health. In the realm of nutrition, ensuring a balanced diet with diverse protein sources is crucial for optimal bodily function. Furthermore, proteins can give rise to bioactive peptides through enzymatic digestion, influencing various physiological processes. As an illustration, certain bioactive peptides, for instance, antidiabetic and anti-obesity peptides generated during protein digestion can interact with the gut microbiota, modulating its composition and activity. These interactions exemplify the intricate relationship between dietary proteins, bioactive peptides, and the gut microbiome, ultimately impacting human health [5,15].

High-quality proteins are vital in increasing satiety and aiding weight management by influencing appetite-regulating hormones, such as leptin and ghrelin [22]. Leptin is produced by adipose tissue and serves as a signal to the brain, particularly the hypothalamus, to communicate the body’s energy stores [30]. When high-quality proteins are consumed, they have been shown to increase the release of leptin. This, in turn, helps to suppress appetite by indicating to the brain that there are sufficient energy reserves, reducing the drive to eat [39].

Conversely, ghrelin, often referred to as the “hunger hormone”, is secreted by the stomach and stimulates appetite. High-quality proteins can effectively reduce ghrelin levels, diminishing the sensation of hunger [52]. This effect is especially pronounced compared to carbohydrates or fats. When protein-rich foods are ingested, they not only slow down gastric emptying, making you fuller for longer, but they also lead to a more gradual and sustained release of energy, preventing the rapid fluctuations in blood sugar that can trigger cravings and overeating [24].

Additionally, high-quality proteins, such as lean meats, fish, eggs, and legumes, are nutrient-dense and can help preserve lean muscle mass during weight loss, which is essential for maintaining a healthy metabolism. As a result, incorporating these proteins into the diet can be a valuable strategy for weight management [53]. They promote a sense of fullness, reduce hunger-inducing hormones, and help preserve muscle mass, collectively contributing to overall improved satiety and more effective weight control. However, it is important to maintain a balanced diet and consult a healthcare professional or nutritionist for personalized advice when embarking on a weight management journey [12].

## 6. Types of Food Proteins 

Food proteins can be broadly categorized into two main types: plant proteins and animal proteins. Plant proteins are obtained from plant-based sources like legumes, nuts, seeds, and grains. Plant proteins are often incomplete, meaning they are deficient in one or more essential amino acids but can be combined to form complete protein sources [54]. Animal proteins, on the other hand, are derived from animal sources, such as meat, poultry, fish, eggs, and dairy products. They are considered complete proteins because they provide all the essential amino acids the human body needs [40]. Both plants and animal proteins are essential components of a balanced diet, and individuals can choose from a variety of sources to meet their nutritional needs and dietary preferences.

## 7. Plant-Based Proteins

Plant-derived proteins have emerged as a potent and versatile resource in managing chronic diseases, offering a promising strategy to meet global health challenges. The increasing prevalence of these diseases and their comorbidities with significant morbidity, mortality, and economic burden highlights the importance and urgency of developing effective prevention, management, and treatment strategies. Plant-derived proteins have gained attention due to their multifaceted nutritional, bioactive, physicochemical, and functional properties and, as such, have found numerous applications among diverse populations and cultures [9]. These proteins, as found in a variety of plants such as fruits, grains, nuts, seeds, and vegetables, usually contains many essential amino acids in addition to providing enough dietary fiber and therefore supports dietary health, tastes, and appetites [55]. Their innate anti-inflammatory and other bioactive properties further enhance their nutritional appeal by potentially reducing oxidative stress and inflammation, key factors in the development and progression of several chronic diseases, especially cancer, CVD, obesity, diabetes, and neurodegenerative diseases [56]. Plant foods that highly express these proteins have shown remarkable efficacy in preventing chronic diseases and improving treatment outcomes. 

Several studies have consistently reported the ability and potential of plant proteins to reduce the risks associated with chronic disease conditions such as heart disease and type 2 diabetes [5,15,44]. These actions are complemented by the fiber contained in foods, which helps regulate blood sugar levels, making them particularly suitable for individuals with diabetes [30]. Plant-based foods can also help prevent weight loss and obesity, as they contain fewer calories and saturated fat while containing more fiber and proteins, which promotes satiety, reduces overall caloric intake, and thereby modulates the effects of chronic diseases [57]. Studies have also shown the various mechanisms by which several plant proteins can influence and modulate disease progression [58]. For example, soy protein is associated with the ability to lower cholesterol levels in vivo making it a beneficial alternative treatment and management approach for individuals with excess cholesterol and arthritis. Other plant proteins have anti-inflammatory properties and can alleviate chronic inflammatory symptoms, such as arthritis [15,48]. Plant-based proteins allow flexibility in digestive strategies to meet specific healthcare needs and provide a comprehensive approach to chronic disease management [59].

Plant proteins have shown exceptional ability to modulate CVD and support and contribute to cardiovascular health [10]. These plant protein-based foods are naturally low in cholesterol, which is known to contribute to atherosclerosis and other heart diseases [25,60]. Also, proteins from specific plants, such as soy protein, have been found to reduce cholesterol, and this is especially valuable for persons with atherosclerosis who consume heart-healthy plant-based diets containing soy proteins [30]. While focusing on high fiber content, including fruits and whole grains, nuts, seeds, and brown rice have been associated with body health, weight maintenance, and therefore, reduction in the risk of developing hypertension, an important component of CVD prevention, plant-based diet with many bioactive proteins and antioxidants also helps with positive effects on cardiovascular health [60]. These antioxidant effects prevent associated oxidative stress and inflammation, which are key factors in developing and progressing CVD and other related chronic conditions, especially several other metabolic syndrome disorders [61].

Food proteins from plant sources have been reported to play important roles in blood sugar regulation, hyperglycemia, and diabetes prevention, control, and management [25]. Plant-based foods, with the exception of a few food crops like legumes and cereal grains, are naturally low in complex carbohydrates, dietary fiber, and simple sugars. These dietary attributes are great for type-2 diabetes control because they help stabilize blood glucose levels and prevent sharp postprandial increases or spikes and falls that can harm overall health [15,22]. In addition, proteins from plant sources added to the diet reduce the rapid release of insulin associated with the consumption of high glycemic index foods [15]. This also provides a constant energy source with no feedback, which can improve glycemic control and reduce insulin resistance, which are major concerns in managing type-2 diabetes [2].

Plant proteins play a crucial role in mitigating the risk of various chronic diseases, including obesity, inflammation, oxidative stress, and certain types of cancer. Plant protein foods contribute to weight management and, therefore, modulate obesity through their high fiber content and ability to induce satiety [62]. Legumes, nuts, and seeds, for instance, offer plant-based protein options that not only promote a feeling of fullness but also provide essential nutrients without excessive caloric intake [35]. This characteristic is particularly beneficial for individuals seeking weight control and overall metabolic health.

Plant protein foods are also associated with anti-inflammatory effects due to the phytochemicals and antioxidants in fruits, vegetables, and other plant sources These compounds aid in combating cellular-level inflammation, thereby reducing the risk of heart disease and vascular issues associated with chronic inflammation [10]. Furthermore, plant protein foods contribute to combating oxidative stress, a factor implicated in various chronic diseases. Antioxidants found in plant-based foods, including vitamins C and E, as well as polyphenols, help neutralize free radicals, thereby protecting cells from damage. This antioxidant defense can have positive implications for conditions associated with oxidative stress, such as neurodegenerative diseases and cardiovascular disorders [63].

Regarding cancer prevention, the diverse array of phytochemicals present in plant-based proteins is thought to play a role in inhibiting the development of certain cancers. For example, cruciferous vegetables contain sulforaphane, which has been studied for its potential anti-cancer properties, especially in relation to breast and prostate cancers. Additionally, the fiber content in plant-based diets is linked to a reduced risk of colorectal cancer [44].

Specific plant protein sources such as legumes, nuts, and seeds exhibit high nutritional value and potential benefits in managing chronic diseases [25,60]. They are very versatile and packed with essential nutrients, making them valuable ingredients in plant-based foods, especially as functional ingredients for developing functional foods and nutraceuticals. Legumes, including beans, corn, and chickpeas, are good sources of plant proteins. 

Nuts and seeds are another source of plant-based proteins that have gained significant recognition for their nutritional, functional and bioactive values. They are high in proteins, healthy fats, vitamins, and minerals, so nuts and seeds such as almonds, walnuts, chia seeds, flax seeds, and hemp seeds have found wide applications in numerous food uses [22]. They are also high in unsaturated fats and are known for their heart health properties, which are useful for reducing LDL-cholesterol. They also contain antioxidants and phytochemicals such as vitamin E, which help reduce inflammation and oxidative stress, important factors in the development and progression of cardiovascular diseases [26]. These are useful for the prevention and management of other chronic diseases, such as obesity, diabetes, cancer [5,64].

The versatility of these plant-based protein sources allows for a wide variety of culinary applications, making it easier for individuals to incorporate them into their diets in various forms, whether in salads, smoothies, or served as ingredients in plant-based foods, such as fruits, nuts, and seeds [9]. This offers a delicious and nutritious approach to enriching a diet rich in plant proteins while also unveiling its health benefits and positive effects on preventing chronic diseases. This unique source of plant-based proteins, therefore, holds great potential for the continued search for their sustainability and effective dietary strategies for the prevention of these chronic diseases [25].

## 8. Animal Proteins

The lean muscles of animals and fishes are high in their contents of proteins, with an excellent balance of essential amino acids profile, therefore, necessitating their importance as not only a nutritious source of high quality proteins but also excellent proteins with functional applications [5,19,31]. Fish proteins offer a myriad of health benefits, particularly in relation to cardiovascular health and brain function. Rich in high-quality, easily digestible proteins, fish provides essential amino acids necessary for various physiological functions [65]. Fish, such as salmon, tuna, and cod, are excellent sources of high-quality proteins, containing essential amino acids crucial for building and repairing tissues in the body. These proteins contribute to muscle development, immune function, and overall body maintenance. Fish proteins are easily digestible, making them a valuable protein source for individuals with digestive sensitivities or those seeking efficient nutrient absorption [12].

Fish is a notable source of high-quality protein, and this attribute makes it particularly valuable in the context of cardiovascular health. The protein found in fish, regardless of the variety, offers numerous advantages for maintaining and promoting heart health [34]. Protein serves as an essential component for the growth, repair, and maintenance of the heart muscle and blood vessels, vital for proper functioning. Moreover, the protein in fish can aid in regulating blood pressure, as it supports the dilation of blood vessels, thereby helping to mitigate the risk of hypertension, a significant cardiovascular risk factor. Additionally, protein’s role in weight management is pivotal, as it contributes to a sense of satiety, assists in appetite control, and thereby influences body weight, which in turn impacts cardiovascular health [12,20,66]. Furthermore, protein-rich foods like fish can assist in stabilizing blood sugar levels, a crucial factor in preventing conditions like type 2 diabetes, known to increase the risk of heart disease. Notably, the protein content in fish also contributes to reducing inflammation, a pivotal factor in the development and progression of CVD. While the holistic cardiovascular benefits of fish are not solely attributable to their protein content, the inclusion of fish in one’s diet, particularly the consumption of fatty fish rich in protein, can be a strategic and healthful choice for promoting better heart health and mitigating the risk of cardiovascular diseases [58].

Fish protein also positively affects cardiovascular health [4]. They are generally low in saturated fat and cholesterol, making them a heart-healthy protein source. Fish proteins also contain bioactive peptides that can exhibit antihypertensive properties lowering blood pressure, which is a major risk factor for hypertension and cardiovascular complications [3]. The therapeutic potential of fish proteins goes beyond prevention. Studies have shown that fish supplementation can improve cardiac function, improve endothelial function, reduce the risk of heart failure, and also contribute to overall cardiovascular health [36,60,64,67].

Different types of fish offer different proteins, making them a perfect choice for different dietary preferences, nutritional needs, and bioactive effects [66,67]. For example, salmon stands out as a protein powerhouse, providing about 22–25 g of protein per 3 ounces (85 g). Usually found in a can, tuna is a high-protein alternative that provides about 22 g of protein per 3-ounce serving. Cod, a mild white fish, has about 15–20 g of protein per serving [60,64]. This difference in protein content allows individuals to choose fish species that match their dietary goals and benefit from the many health benefits associated with fish consumption, including improved cardiovascular health and essential nutrients such as vitamins and mineral resources [28].

Dairy proteins, derived from milk and its products, represent a valuable and complete source of essential amino acids necessary for various bodily functions. Dairy proteins are crucial for muscle development and repair and contribute to overall bone health due to the presence of calcium and other essential minerals in dairy products [12,13]. Additionally, the bioactive peptides found in dairy proteins may have various health-promoting properties, including potential immune system benefits [13,31,44]. While dairy proteins are nutrient-dense and offer a range of health advantages, it is essential to consider individual tolerances and dietary preferences when incorporating them into a balanced and diverse diet.

Dairy foods, rich in protein, contribute to muscle preservation, glycemic control, weight management, and bone health, collectively promoting overall well-being and reducing cardiovascular risk factors [61]. First and foremost, the protein in dairy products is a valuable resource for preserving and repairing muscle tissue. These proteins supply essential amino acids necessary for muscle maintenance, and their consumption supports the health and function of muscles, which, in turn, are integral to cardiovascular health, as a robust musculature supports the circulatory system’s efficiency [31,44].

Regarding glycemic control, dairy proteins exhibit benefits by modulating blood sugar levels. The consumption of dairy products can lead to a slower, more gradual release of glucose into the bloodstream, helping to prevent rapid spikes and crashes in blood sugar levels [67]. This aids in mitigating the development of insulin resistance and assists in managing conditions like type 2 diabetes, which are closely linked to cardiovascular risk.

Weight management is another area where dairy proteins excel. Their satiating effect helps curb appetite and prolong the feeling of fullness, contributing to calorie control and assisting individuals in maintaining healthy body weight [68]. Effective weight management is pivotal for cardiovascular health, as excess body weight can increase the risk of heart disease and related conditions [35].

Milk is a valuable source of whey casein and protein, and it offers an easy and nutrient-dense way to incorporate this high-quality protein into the daily diet. Milk, the main dairy component, is about 80% casein and 20% whey, with a balanced protein blend as an alternative [69]. Cheeses such as cottage cheese and ricotta are notable for their casein content, which aids muscle retention and appetite suppression [70]. Greek yogurt, rich in whey and casein proteins, offers a creamy and high-protein option suitable for muscle function and flavor enhancement. Additionally, whey protein supplements from dairy products are readily available for enhanced muscle maintenance and recovery [70]. They provide essential nutrients and cater to various dietary preferences to achieve desired nutritional and health goals, including muscle preservation, glycemic control, weight management, bone health, and numerous other functions [68].

Whey protein is a rapidly digestible source of proteins rich in essential amino acids, especially leucine, which plays an important role in muscle protein synthesis, such as sarcopenia and cancer-related muscle wasting, and in regulating blood sugar [71]. Positive effects have been shown in the modulation of type 2 diabetes, making it useful for type 2 diabetics. It can increase insulin sensitivity, improve glucose metabolism, and reduce the risk of insulin resistance and complications related to diabetes [67].

Casein protein has been associated with slow digestion and prolonged release of amino acids, keeping amino acids supplied for longer periods of time [72]. These properties can regulate appetite control while creating a sense of fill, making it useful for weight management in individuals with obesity-related chronic diseases [45]. Also, casein protein is associated with reduced cardiovascular risk factors, including hypertension [73]. According to a recent study, peptides derived from casein may have anti-hypertensive properties by inhibiting enzymes responsible for hypertension, especially renin and angiotensin II converting enzyme (ACE) involved in the renin-angiotensin-aldosterone (RAAS) pathway [68].

Both whey and casein proteins provide immune-boosting properties due to their rich content of essential amino acids and functional peptides [48]. Therefore. these proteins can support immune function, which is especially important for individuals dealing with chronic diseases due to compromised immune systems. Additionally, these proteins play a role in maintaining bone health, which is important for individuals with conditions such as osteoporosis. The high calcium content of dairy proteins, including casein, helps build bone strength, while the amino acids in these proteins promote the stimulation of collagen synthesis and bone health; therefore, they reduce the risk of bone fracture [51].

## 9. Functional Ingredients in Chronic Disease Management

Functional ingredients play a pivotal role in the field of chronic disease management, offering specific health benefits beyond basic nutrition [74]. These ingredients, often derived from various foods, are recognized for their potential to promote well-being, and mitigate the risk or severity of chronic conditions. From antioxidants to fiber and bioactive compounds, functional ingredients contribute to a holistic approach to health, emphasizing nutrition’s preventive and therapeutic aspects [6].

Functional ingredients refer to bioactive compounds within foods that provide health benefits beyond their nutritional value [75]. These ingredients have specific physiological effects on the body and are often associated with preventing or managing chronic diseases. Examples include antioxidants, probiotics, and phytochemicals, each contributing unique properties that support overall health [76]. Functional ingredients positively impact chronic disease management by targeting specific disease development or progression mechanisms. Antioxidants, for instance, help combat oxidative stress, a key factor in various chronic conditions. Probiotics support gut health by influencing the immune system and potentially reducing inflammation [71]. The diverse range of functional ingredients provides a tailored and preventive approach to chronic disease management, aligning with the growing emphasis on food as medicine.

Food proteins, acting as functional ingredients, play intricate roles in promoting health and managing chronic diseases across various dietary contexts [77]. Whey protein, derived from milk, is renowned for its diverse bioactive peptides. These peptides have immunomodulatory effects, influencing immune responses and potentially contributing to the prevention of infections and inflammatory conditions. Furthermore, whey’s high leucine content makes it a valuable component in muscle health, aiding in recovery after exercise and potentially mitigating age-related muscle loss [78].

Plant-based proteins, found in legumes, nuts, seeds, and grains, contribute various health benefits. Beyond being excellent sources of essential amino acids, plant proteins bring a plethora of bioactive compounds [79]. Polyphenols, flavonoids, and other antioxidants present in plant proteins exhibit anti-inflammatory properties, potentially reducing the risk of chronic inflammatory diseases. The fiber content in these proteins supports digestive health, helps regulate blood sugar levels, and assists in weight management—an essential aspect of preventing and managing conditions like diabetes and obesity [80].

Although often associated with omega-3 fatty acids, fish proteins also contain bioactive peptides. These peptides have demonstrated cardiovascular benefits, including the potential to lower blood pressure and improve lipid profiles. The combination of proteins and omega-3 fatty acids in fish contributes to heart health, showcasing the multifaceted nature of fish as a functional ingredient [29].

In the broader context of functional foods, incorporating these diverse protein sources exemplifies the synergy between essential nutrients and specific health-promoting properties. The unique attributes of food proteins contribute to the provision of vital amino acids and the nuanced and targeted support of various physiological functions [81]. As functional foods continue to evolve, understanding and harnessing the potential of food proteins offers promising avenues for personalized dietary interventions in chronic disease prevention and management [75].

## 10. Conclusions

In conclusion, the role of food proteins as functional ingredients in the management of chronic diseases is multifaceted and promising. From whey protein’s immunomodulatory effects to plant-based proteins’ antioxidant and anti-inflammatory properties and the cardiovascular benefits associated with fish proteins, these functional ingredients contribute significantly to holistic health. The diverse bioactive compounds present in food proteins not only provide essential amino acids for bodily functions but also offer specific health-promoting benefits. The incorporation of these proteins into functional foods aligns with the evolving paradigm shift of using nutrition as a preventive and therapeutic tool in chronic diseases management. As research continues to unveil the intricate relationships between food proteins and physiological well-being, there is a growing appreciation for the potential of personalized dietary interventions. The dynamic interplay between essential nutrients and bioactive compounds within food proteins underscores their vital role in promoting overall health and supporting individuals in the prevention and management of chronic diseases.
